# Optical coherence tomography angiography–guided vs indocyanine green angiography–guided half-dose photodynamic therapy for acute central serous chorioretinopathy: 6-month randomized trial results

**DOI:** 10.1007/s00417-023-06147-5

**Published:** 2023-06-22

**Authors:** Siying Li, Linqi Zhang, Jiyang Tang, Zongyi Wang, Jinfeng Qu, Mingwei Zhao

**Affiliations:** Department of Ophthalmology, Beijing Key Laboratory of Diagnosis and Therapy of Retinal and Choroid Diseases, College of Optometry, Peking University People’s Hospital; Eye Diseases and Optometry Institute; Peking University Health Science Center, No. 11 South Avenue of XiZhiMen, 100044 Xi Cheng District, Beijing, People’s Republic of China

**Keywords:** OCTA-guided, ICGA-guided, Acute CSC, PDT

## Abstract

**Purpose:**

This study aimed to compare the anatomic and functional results of optical coherence tomography angiography (OCTA)-guided half-dose photodynamic therapy (PDT) versus indocyanine green angiography (ICGA)-guided PDT in eyes with acute central serous chorioretinopathy (CSC).

**Methods:**

One hundred and thirty-one eyes of 131 patients with acute central serous chorioretinopathy (CSC) were recruited, and randomly assigned to the OCTA-guided group and ICGA-guided group. The primary outcome measures were the rates of complete subretinal fluid (SRF) resolution at 1 month, 3 months, and 6 months. The secondary outcomes included best-corrected visual acuity (BCVA), central retinal thickness (CRT), choroidal capillary flow deficit density at each scheduled visit, and recurrence rate of SRF at 3 months and 6 months.

**Results:**

There were 110 eyes that finished the follow-up, with 56 eyes in the OCTA-guided group and 54 eyes in the ICGA guided group. OCTA-guided PDT was demonstrated to be noninferior to ICGA-guided PDT for SRF resolution rate at 1 months and 6 months (*P* = 0.021 and *P* = 0.037), but not at 3 months for acute CSC (*P* = 0.247). The average CRT of the ICGA-guided group was significantly lower than that of the OCTA-guided group at 3-month visit (*P* = 0.046), but no significant difference was found between them at the 1-month and 6-month visits (*P* = 0.891 and 0.527). There was no significant difference between the two groups for BCVA (*P* = 0.359, 0.700, and 0.143, respectively) and the deficit area on CC (*P* = 0.537, 0.744,and 0.604, respectively) at 1, 3, and 6 months.

**Conclusion:**

OCTA may replace ICGA to guide PDT for the treatment of acute CSC and their follow-up.

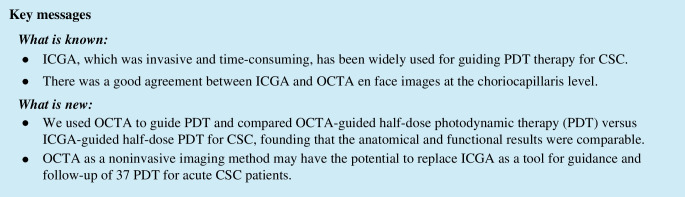

## Introduction 

Central serous chorioretinopathy (CSC) is a chorioretinal disease characterized by subretinal fluid (SRF) on optical coherence tomography (OCT), with focal or diffuse leakage on fundus fluorescein angiography (FFA) and choroidal vascular hyperpermeability (CVH) on indocyanine green angiography (ICGA) [1]. Acute CSC is generally self-limiting with a favorable outcome, but 30–50% of patients may experience permanent vision loss or recurrent within one year and 5% of patients may develop chronic CSC, which result in retinal pigment epithelium (RPE) damage and progressive decline of visual acuity [2, 3]. Photodynamic therapy (PDT) with half dose of verteporfin has been proved to be effective in the treatment of acute CSC [4, 5]. However, ICGA, which is required to locate the treating area before PDT, is invasive and ICGA-related adverse events have been reported as potential drawback of PDT [6].

Optical coherence tomography angiography (OCTA) is a novel non-invasive, time-saving, high-resolution imaging technic that can clearly depict and quantitatively analyze the microvascular abnormalities of different layers of retina and choroid. Previous studied have found good agreement between ICGA and OCTA en face images at the choriocapillaris (CC) level [7–9]. Therefore, we hypothesized that OCTA may replace ICGA to guide the positioning of PDT for CSC. Our pilot study observed 23 eyes with acute CSC and found that OCTA-guided PDT was noninferior to ICGA-guided PDT in terms of subretinal fluid (SRF) resolution rate at 3-month follow-up [10]. In this study, we enrolled more patients with longer follow-up to compare the therapeutic effects of OCTA-guided and ICGA-guided PDT.

## Materials and methods

### Design

This study was a prospective, single-center, double masked, randomized, controlled clinical trial study of OCTA-guided versus ICGA-guided PDT for the treatment of acute CSC which was registered at Clinical Trials. gov (NCT03497000). This study was approved by the Ethics Committee Review Board of Peking University People’s Hospital (2020PHB250-01). Written informed consents were signed by all patients before PDT. All procedures adhered to the tenets of the Declaration of Helsinki. Patients who were diagnosed with acute CSC in Peking University People’s Hospital from November 2019 to October 2020 were screened for eligibility.

### Inclusion criteria

The patients were consecutively enrolled in our study when the following inclusion criteria were fulfilled: (1) age between 18 and 50 years old; (2) treatment naïve CSC with symptoms less than 6 months or asymptomatic patients with medical record documentation proving the presence of SRF; (3) presence of SRF involving the fovea on OCT; (4) evidence of focal fluorescein leakage on FFA; (5) CVH detected by ICGA; (6) evidence of hyper-reflective areas on OCTA en face image at the CC level with image quality > 6.

### Exclusion criteria

The exclusion criteria were as follows: (1) previous PDT, focal photocoagulation, intravitreal injections, or ocular surgery; (2) chronic CSC with secondary CNV on OCTA; (3) other macular abnormalities such as age-related macular degeneration or polypoidal choroidal vasculopathy; (4) other disease that may cause choroidal thickening, such as scleritis, choroidal inflammation, choroidal neoplasms; (5) other disease that may cause retinal vascular leakage, such as uveitis, diabetic retinopathy; (6) conditions that may cause poor OCTA signaling, such as high myopia, cataract, or nystagmus; (7) systemical or topical steroid usage in the last 6 months or during the follow-up; (8) patients who cannot tolerate FA or ICGA.

### Randomization and masking

Patients were randomized at a ratio of 1:1 into the OCTA group and ICGA group. The randomization sequence was generated using a computerized randomization table. Allocation was performed before PDT and all patients, examiners, PDT investigators, and graders were masked to the assignment.

### Baseline and follow-up visit

All patients underwent comprehensive ophthalmologic examinations at baseline, 1 month, 3 months, and 6 months after PDT including best-corrected visual acuity (BCVA), intraocular pressure (IOP), slit-lamp biomicroscopy, dilated ophthalmoscopy, SD-OCT and OCTA (CIRRUS HD-OCT Model 5000, Carl Zeiss Meditec), FA (FF 450 plus, Carl Zeiss Meditec AG), and ICGA (Heidelberg Engineering, Heidelberg, Germany). For OCTA, 6 × 6 mm scan centered on the fovea were performed. In cases SRF were beyond the scanning field, additional 6 × 6 scans were obtained to create a larger montage to ensure that all SRF were included. Manually correction was performed by using built-in OCTA software if there were automated segmentation error.

### Intervention

In OCTA group, the hyper-reflective area on en face OCTA image at CC level was identified as treating area. In ICGA group, the area of CVH on ICGA which was related to the leakage on FA and SRF on OCT was identified as treating area. The treating area was outlined on OCTA or ICGA images and then registered and marked on the color fundus photograph (CFP) to guide PDT laser spot. PDT was all performed by the same retinal specialist (ZMW) who was given only the CFP with pre-outlined treating area. After measurement of body weight and estimation of body surface area, 3 mg/m^2^ of verteporfin were infused, and a PDT laser with a 689-nm wavelength and power of 50 J/cm^2^ was used to delivered the treatment to the target area for 83 s via PDT laser lens (Volk Optical Inc., USA). Patients were instructed to avoid bright lights and sunlight for at least 48 h after PDT.

### Outcomes measurements

The primary outcome was the proportion of eyes with complete absorption of SRF on OCT at each follow-up visit. The secondary outcomes included BCVA, central retinal thickness (CRT), choroidal capillary flow deficit density at each scheduled visit, and recurrence rate of SRF at 3 months and 6 months.

**Fig. 1:**
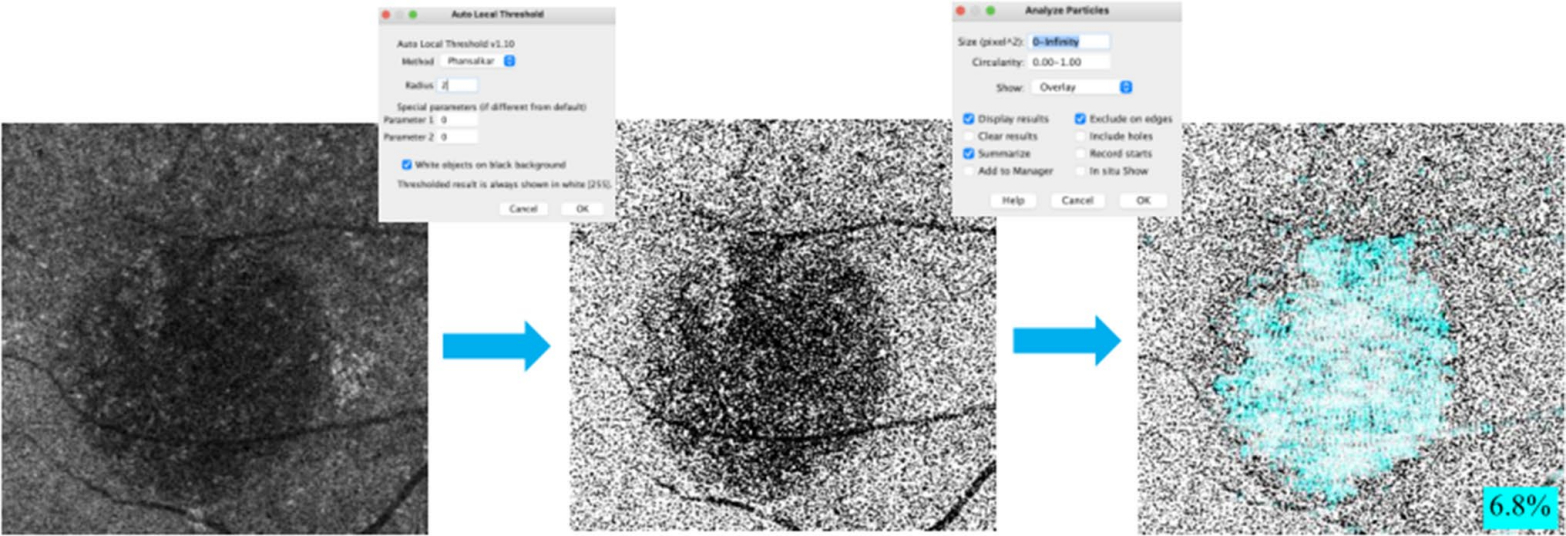
Binarizing of choriocapillaris flow imaging and measurement of flow deficit density (FDD) by using Image J

BCVA was converted from decimal to logMAR for statistic purpose. The measurement of CRT was performed by measuring the length between internal limiting membrane (ILM) and Bruch’s membrane manually using the build-in tools in OCT instrument. The measurement of choriocapillaris flow deficit density (FDD) was as follows: on OCTA images (the percentage of the missing area of vessel flow density in the total scanned area) are as follows: the 6 × 6 mm en face OCTA images (450 × 450 pixels) at CC layer were imported into the Image J software (version 1.53a). After adjusting them to 8 bit images, automatic local thresholding was done with Phansalkar method using radius of 2 pixels to binarize the images. Then, we selected “analyze particles” command under “analyze” menu, set size to 0-infinity, circularity to 0–1 and selected showing overlay [11]. By doing this, the software marked the choriocapillaris flow deficit area with blue and automatically calculated FDD (the percentage of the flow deficit area divided by total scanned area). We exported these images and recorded FDD value (Fig. 1).

### Statistics

The sample size was calculated based on the following assumptions for the primary efficacy end point: a 2-sided significance level of 5%, a power of 80%, a noninferiority margin of 10%, and an expected SRF resolution rate of PDT for the two treatment groups of 97%. Under these assumptions, the estimated required sample size should be at least 46 in each group.

All statistical analyses were performed with the SPSS software (IBM SPSS Statistics 19.0). Comparisons of baseline demographics and clinical data were made between the 2 groups using the 2-tailed *t* test or the Wilcoxon signed rank test for continuous variables and Pearson *χ*^2^ test or Fisher exact test for binary variables. *P* value of 0.05 was considered to be statistically significant. A noninferiority test was applied to compare the complete SRF absorption rate on OCT at 1, 3, and 6 months between OCTA-guided and ICGA-guided groups with a noninferiority margin of 10%.

## Results

### Baseline demographics

**Fig. 2:**
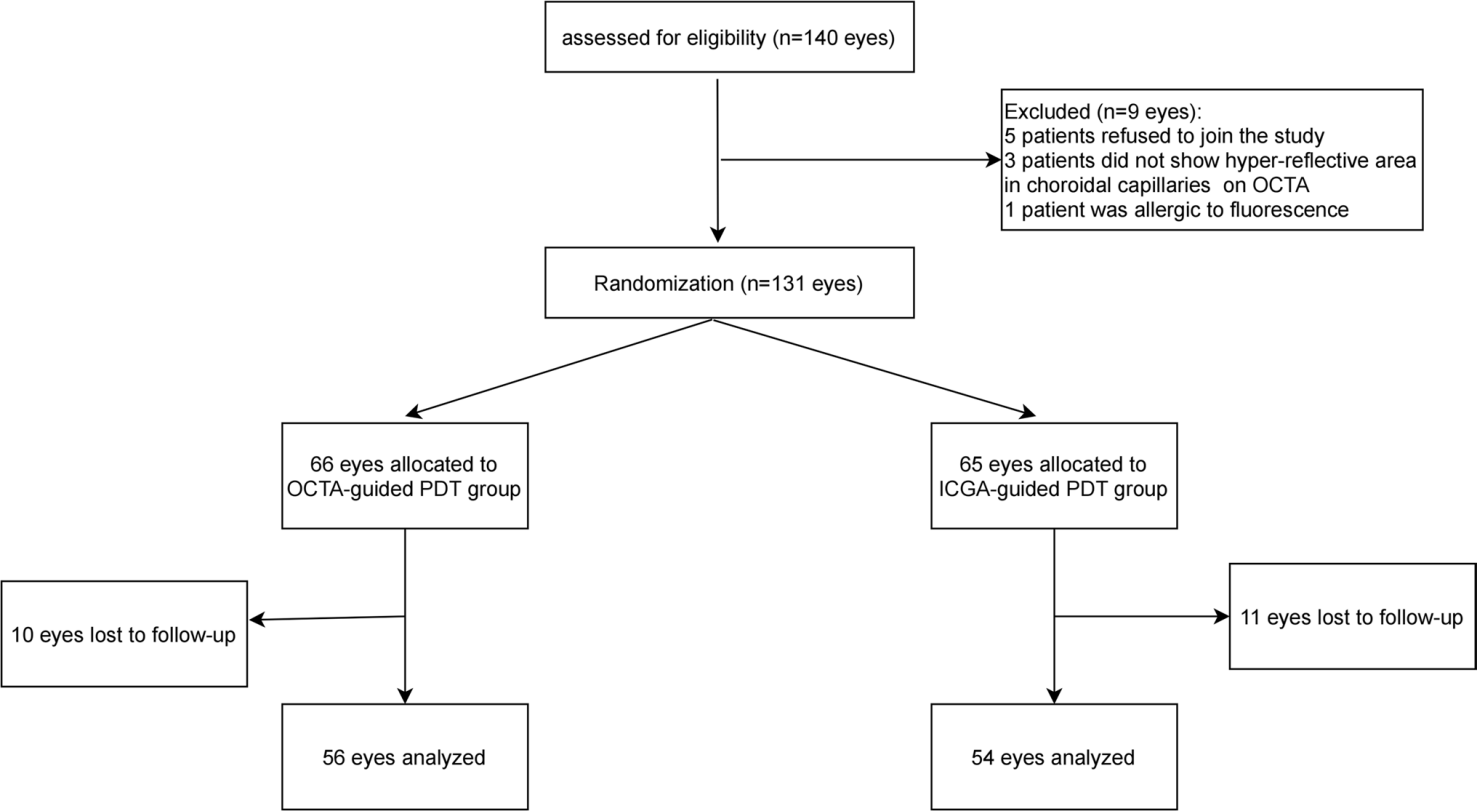
Flowchart showing the distribution of the study population

A total of 140 eligible eyes were screened, and 131 eyes were assigned randomly into two groups: 66 eyes in the OCTA group and 65 eyes in the ICGA group. Twenty-one eyes were lost to follow-up, and 56 eyes in the OCTA group and 54 eyes in the ICGA group were analyzed finally in the study (Fig. 2). Baseline characteristics and demographics of 110 included eyes in both groups are listed in Table 1. There were no statistically significant differences in age, sex, duration of symptom, BCVA, IOP, CRT, the proportion of eyes with hypertension or PED, and spot diameter between the two groups (Table 1).

**Table 1:** Baseline characteristics of OCTA group and ICGA group

Characteristics	OCTA group (*n* = 56)	ICGA group (*n* = 54)
Age (year)	43.7 ± 6.9	41.2 ± 7.8
Sex (male/female)	38/18	45/9
Duration of symptom (m)	2.5 ± 1.9	2.3 ± 2.0
Hypertension, *n* (%)	5 (9.8)	6 (12.0)
BCVA (logMAR)	0.39 ± 0.32	0.38 ± 0.31
IOP (mmHg)	15.61 ± 3.19	14.78 ± 3.27
CRT (um)	367.2 ± 170.1	330.8 ± 135.7
PED, *n* (%)		
Yes	36 (64.3)	30 (55.6)
No	20 (35.7)	24 (44.4)
Spot diameter (um)	3925.8 ± 719.2	3681.8 ± 603.0

*OCTA* optical coherence tomography angiography, *ICGA* indocyanine green angiography, *logMAR* logarithm of minimum angle of resolution, *BCVA* best-corrected visual acuity, *IOP* intraocular pressure, *CRT* central retinal thickness, *PED* pigment epithelial detachment

### Resolution of SRF on OCT

**Fig. 3:**
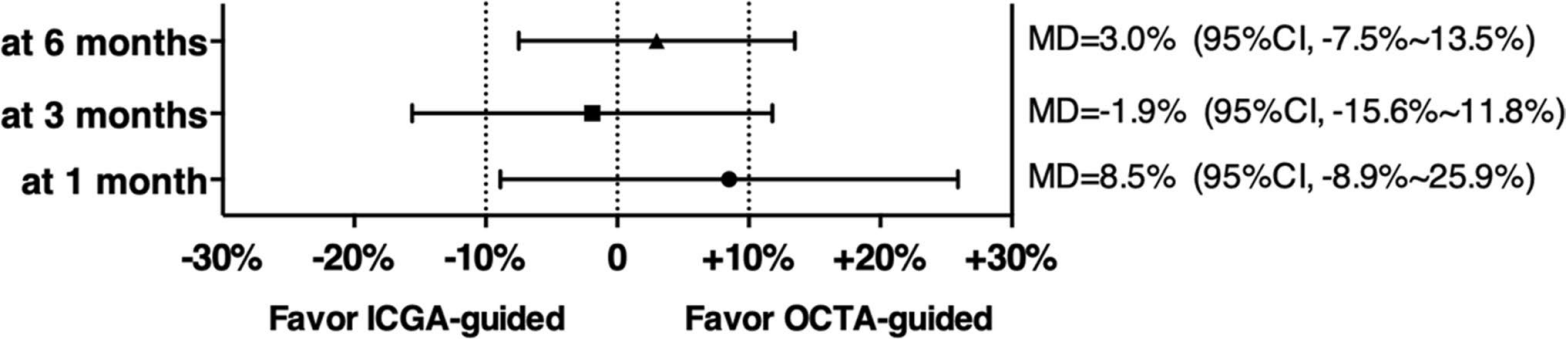
Non-inferiority test of OCTA-guided PDT to ICGA-guided PDT for complete SRF resolution rate at 1, 3, and 6 months after treatment

**Fig. 4:**
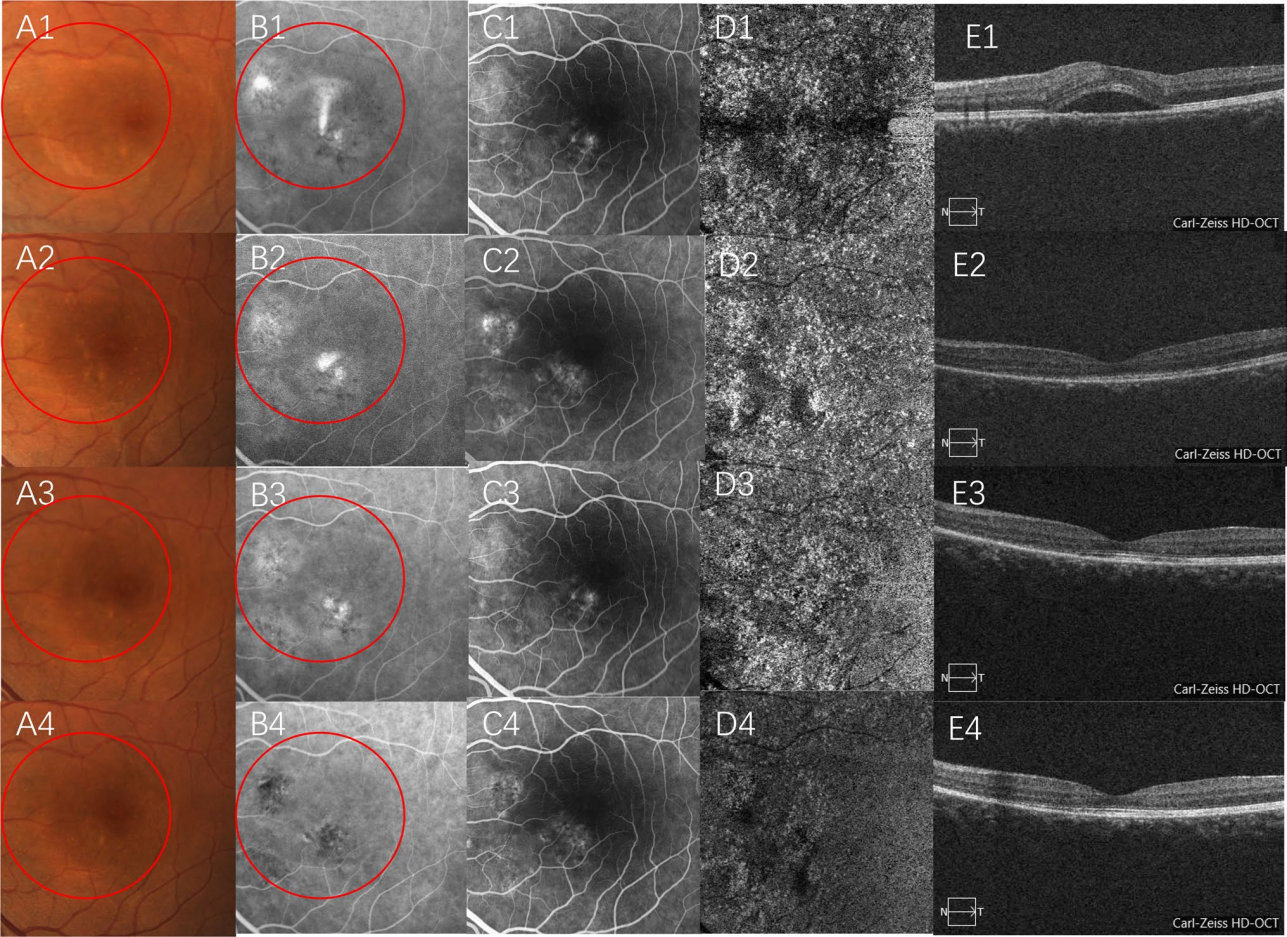
Images of a 23-year-old man with acute CSC treated with ICGA-guided PDT. The patient companied of blurred vision of the left eye for 1 week. CFP, ICGA, and FFA images, 6 × 6 mm en face OCTA image at the level of the choriocapillaris, B scan of the structural OCT before PDT (A1–E1), 1 month after PDT (A2–E2), 3 months after PDT (A3–E3), and 6 months after PDT (A4–E4). Red circles indicate the PDT spot area. Note the SRF was completely resolved at 1 month after PDT (CSC, central serous chorioretinopathy; OCTA, optical coherence tomography angiography; PDT, photodynamic therapy; CFP, color fundus photograph; ICGA, indocyanine green angiography; FFA, fundus fluorescein angiography; SRF, subretinal fluid)

**Fig. 5:**
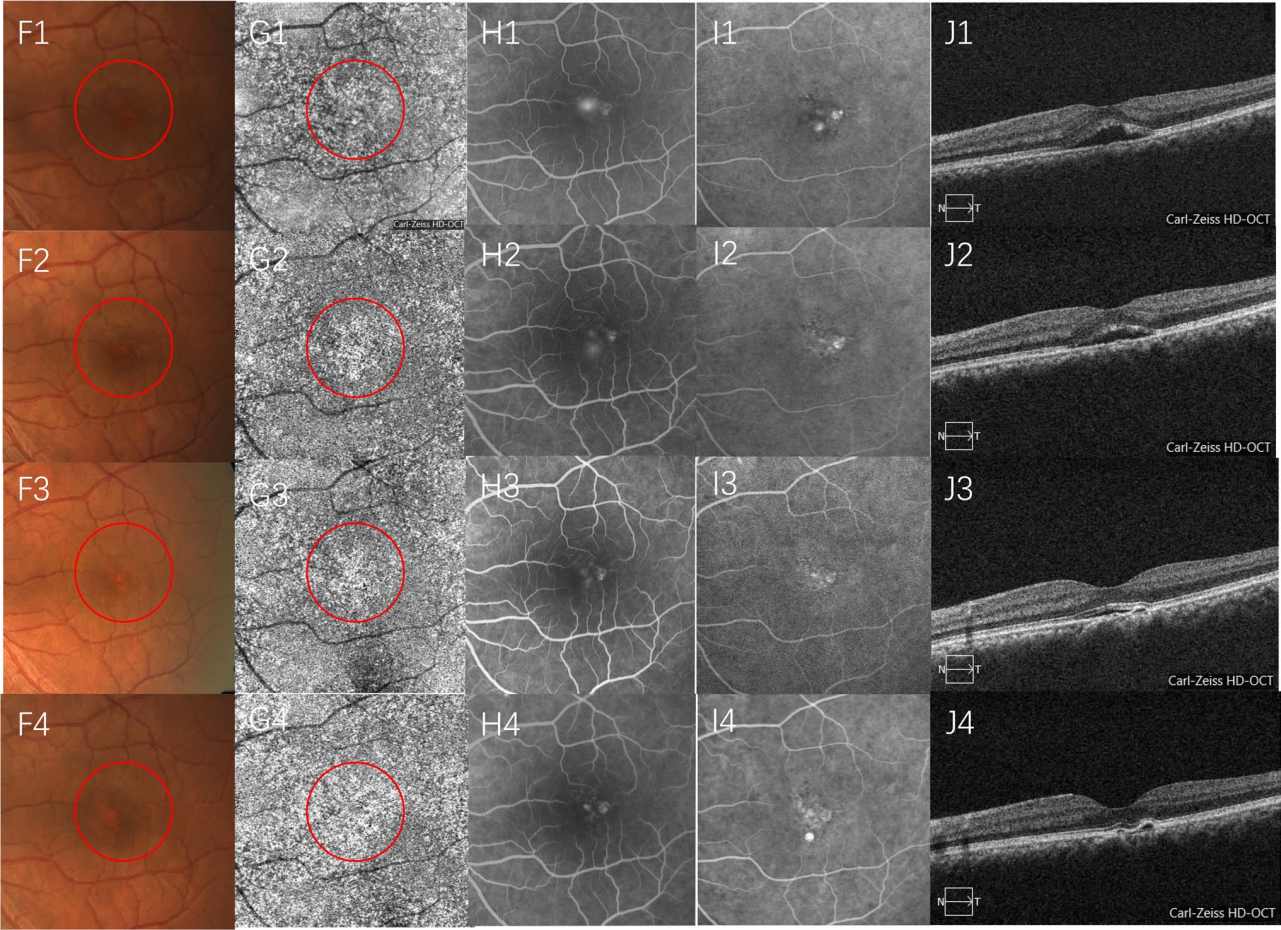
Images of a 42-year-old man with acute CSC treated with OCTA-guided PDT. CFP, ICGA, and FFA images, 6 × 6 mm en face OCTA image at the level of the choriocapillaris, B scan of the structural OCT before PDT (A1–E1), 1 month after PDT (A2–E2), 3 months after PDT (A3–E3), and 6 months after PDT (A4–E4). Red circles indicate the PDT spot area. Note the SRF was partially resolved at 1 and 3 months after PDT and completely resolved with a small serous PED appeared at 6 months after PDT. The leakage on FFA and ICGA partially resolved at 1 and 3 months after PDT. The en face OCTA image at the level of the choriocapillaris hyper-reflective area on OCTA also gradually disappeared during the 6 months of follow-up (CSC, central serous chorioretinopathy; OCTA, optical coherence tomography angiography; PDT, photodynamic therapy; CFP, color fundus photograph; ICGA, indocyanine green angiography; FFA, fundus fluorescein angiography; SRF, subretinal fluid)

At 1-month follow-up, 76.5% eyes in OCTA group and 68.0% in ICGA group had complete SRF resolution. The mean difference in complete SRF resolution rate between the two groups (OCTA group minus ICGA group) was 8.5% (95% CI − 8.9–25.9%). The proportion of eyes with complete SRF absorption was 86.7% in the OCTA group and 88.6% in the ICGA group at 3 months, 95.3% and 92.3% at 6 months. The mean difference of them was − 1.9% (95%CI − 15.6–11.8%) and 3.0% (95%CI − 7.5–13.5%) at 3 and 6 months, respectively. The inferiority of OCTA-guided PDT to ICGA-guided PDT was demonstrated at 1 month and 6 months (*P* = 0.021 and *P* = 0.037, respectively), but not at 3 months for acute CSC patients (*P* = 0.247) (Table 2; Fig. 3). The representative cases in the ICGA-guided and OCTA-guided PDT group were showed in Figs. 4 and 5.

**Table 2:** Proportions of eyes with complete SRF resolution on OCT between two groups

SRF resolution	OCTA group	ICGA group
1 month, n (%)	39/51 (76.5)	34/50 (68.0)
3 months, n (%)	39/45 (86.7)	39/44 (88.6)
6 months, n (%)	41/43 (95.3)	36/39 (92.3)

*SRF* subretinal fluid, *OCT* optical coherence tomography, *OCTA* optical coherence tomography angiography, *ICGA* indocyanine green angiography

### Recurrence rate of SRF

The recurrence rate of SRF at 3 months and 6 months after PDT was 13.3% and 16.3% in the OCTA group versus 11.4% and 17.9% in the ICGA group. There was no statistical difference in recurrence rate of SRF between the two groups at 3 months and 6 months (*P* = 0.778 and 0.841) (Table 3).

**Table 3:** Proportions of SRF recurrence on OCT between two groups

SRF recurrence	OCTA group	ICGA group	*P* value
3 months, n (%)	6/45 (13.3)	5/44 (11.4)	0.778
6 months, n (%)	7/43 (16.3)	7/39 (17.9)	0.841

*OCT* optical coherence tomography, *OCTA* optical coherence tomography angiography, *ICGA* indocyanine green angiography

### Changes of CRT, BCVA

**Fig. 6:**
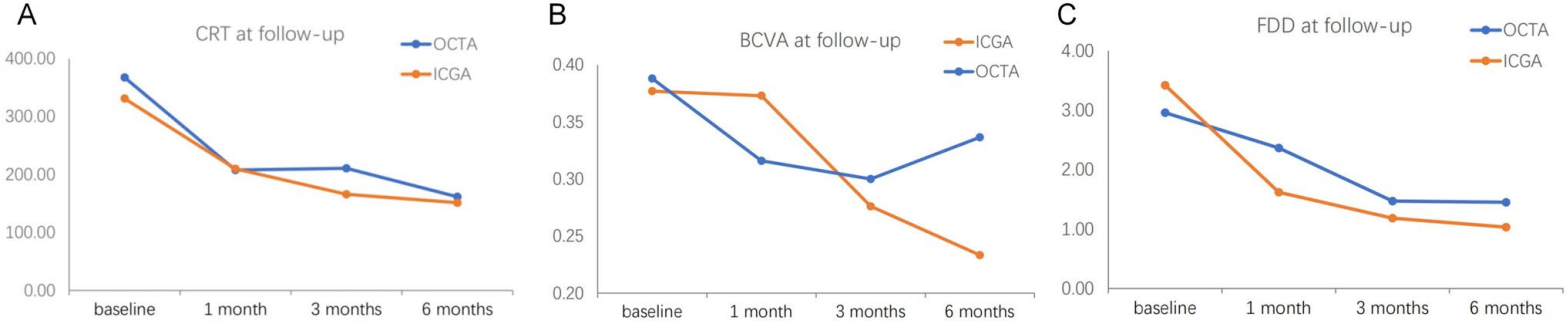
**A** Comparison of CRT at each visit between two groups. **B** Comparison of Log MAR BCVA at each visit between two groups. **C** Comparison of FDD at CC level on 6 × 6 mm en face OCTA image at each visit between two groups

After PDT, the average CRT decreased from 367.2 ± 170.1 μm at baseline to 207.6 ± 90.3 μm at 1 month, 210.5 ± 123.5 μm at 3 months, and 161.5 ± 69.5 μm at 6 months in the OCTA group. In the ICGA group, the average CRT decreased from 330.8 ± 135.7 μm to 209.7 ± 79.1 μm at 1 month, 165.8 ± 67.0 μm at 3 months, and 151.3 ± 73.0 μm at 6 months. The average CRT of the ICGA group was significantly lower than that of the OCTA group at 3-month visit (*P* = 0.046), but no significant difference was found between them at the 1-month and 6-month visits (*P* = 0.891 and 0.527, respectively) (Fig. 6).

The average logMAR BCVA after PDT was 0.32 ± 0.27 at 1 month, 0.30 ± 0.30 at 3 months, and 0.34 ± 0.31 at 6 months in the OCTA group versus 0.37 ± 0.32, 0.28 ± 0.29, and 0.23 ± 0.28 in the ICGA group, respectively. There was no significant difference between the two groups at each visit (*P* = 0.359, 0.700, and 0.143) (Fig. 6).

### Change of choriocapillaris flow deficit density

**Fig. 7:**
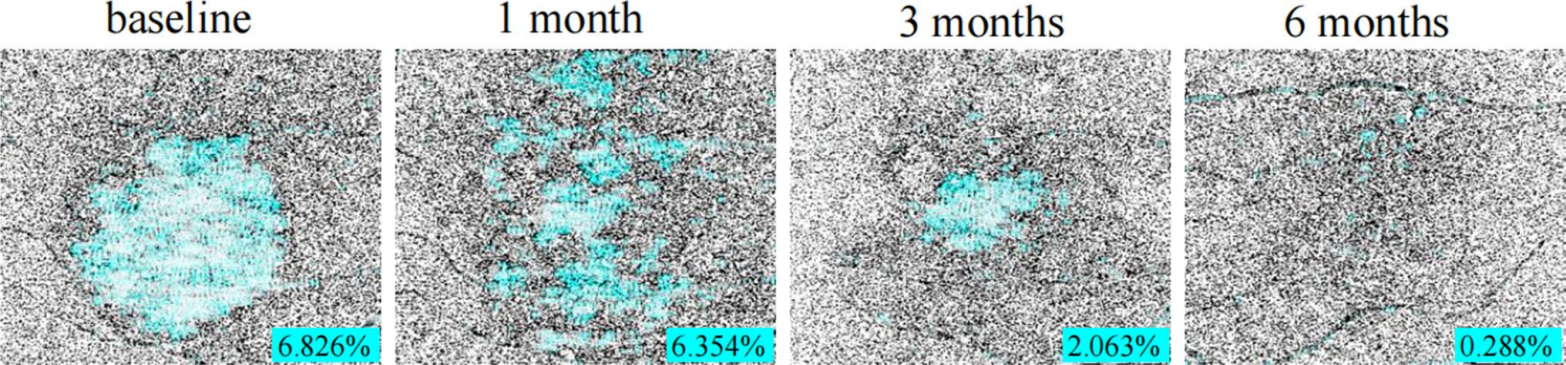
The change in choroicapillaris flow deficit density (FDD) after half dose PDT calculated by using binarized OCTA image at CC level throughout follow-up period

At baseline, choriocapillaris flow deficit area present as granulated low reflective area on en face OCTA image at CC level was observed in all eyes. After PDT, the deficit area on CC showed gradual reduction during the follow-up in both the OCTA and ICGA groups. The mean FDD decreased from 2.96 ± 3.72% at baseline to 2.36 ± 4.00% at 1 month, 1.47 ± 2.56% at 3 months, and 1.45 ± 2.32% at 6 months in the OCTA group, and from 3.42 ± 5.19% to 1.62 ± 3.00% at 1 month, 1.18 ± 0.84% at 3 months, and 1.03 ± 0.83% at 6 months in the ICGA group. There was no significant difference between the two groups at each follow-up visit (*P* = 0.537, 0.744, and 0.604, respectively) (Figs. 6 and 7).

### Safety

No systemic adverse events associated with verteporfin infusion were observed in our study. We did not observe RPE tear or secondary CNV during the follow-up period. We found choriocapillaris perfusion was markedly decreased at the site of the PDT spot in only 1 patient at 1, 3, and 6 months after PDT and the severity of choriocapillaris ischemia decreased with the extension of follow-up time.

## Discussion

As a common disease worldwide especially in Asia, CSC is now believed belong to pachychoroid spectral disease. Although lack of animal model prevents understanding the basic feature of CSC, development in multimodal imaging in recent years provides us new insight to the pathogenesis of CSC.

From the multifocal CVH on ICGA of CSC, many investigators proposed the theory that this hyperpermeability and consequently dysfunction of choriocapillaris–RPE complex eventually causes neurosensory retinal detachment in CSC. CVH can attributed to multiple factors such as RPE degeneration, possible infection, choriocapillaris ischemia, and congestion from direct compression of underlying abnormally dilated choroidal vessels, high levels of corticosteroid and catecholamines, coagulation, or circulating antigen–antibody complexes in vessels [2, 12]. The mechanism of PDT to treat CSC remains incompletely understood. It has been postulated that it can reduce choroidal overflow and CVH, remodeling choroidal choriocapillaris, without damaging the overlying RPE and photoreceptors [13, 14]. Half-dose PDT is widely used as a standard treatment for CSC, because it was been proved to be as effective as full-dose PDT with low risk of PDT induced choroidal hypoperfusion and secondary atrophy [15]. Laser spot of PDT was commonly applied to cover the hyperpermeability area as determined by ICGA [16].

With the development of OCTA, our understanding of CSC had been refined. Costanzo and Chan et al. have reported abnormal appearance of OCTA image at CC level and found it was related to CVH on ICGA [8, 17]. Our previous study showed three types of anomalies in en face OCTA images at CC level and the area of type A abnormality which present as coarse granulated high reflective area was statistically larger than that of CVH in macular region on ICGA although they corresponded well in most of the cases with a mean Jaccard index larger than 0.8 [9]. Since insufficient cover of lesion area from laser spot may lead to fail of SRF resolution, we designed this study to compare the efficacy of PDT under the guide of these two different imaging modalities.

From the anatomic point of view, OCTA-guided PDT was proved to be noninferior to ICGA-guided PDT at 1 month and 6 months in this study. The noninferiority at 3 months may require larger number of subjects to be tested in future study. Although similar recurrence rate of SRF was found between the two groups at 3 months and 6 months, eyes in the OCTA-guided group showed larger mean CRT at 3 months comparing to the ICGA-guided group. This may be explained by thicker SRF in recurrent eyes in the OCTA-guided group at 3-month visit.

From the functional point of view, both groups showed gradual improvement of BCVA by 3 months. Although no significant difference of BCVA was found between the two groups at each visit, we noticed that mean BCVA dropped from 0.30logMAR at 3 months to 0.34logMAR at 6 months in the OCTA-guided group. One explanation is BCVA was affected by multiple factors such as duration of SRF before treatment, EZ integrity, persistent choriocapillaris ischemia, thinning of outer nuclear layer, and range of RPE damage, other than persistent SRF itself [18]. Another possible reason is that there were more eyes accept secondary half-dose PDT in the OCTA-guided group (5 vs 1, Fisher exact test, *P* = 0.206) and more session of PDT was reported to be a risk factor of macular atrophy and poor visual acuity.

Recent studies of ICGA in CSC reported by Cheung and Spaide found delay and pulsatile filling of choroidal veins and the leakage seen in ICGA occurred in regions at or near the intervortex venous anastomoses. They postulate that the choriocapillaris ischemia and congestion are probably not from dilated choroidal vessels but rather from a more generalized increase outflow pressure of choroidal vein [19]. Spaide also pointed out that PDT cannot change the underlying choroidal veins outflow obstruction, so that recurrence of SRF may occur [19]. In our study, the recurrence rate of SRF was around 11–13% at 3 months and 16–18% at 6 months after PDT. According to the theory of Spaide, the recurrent rate may relate to other underlying factors other than laser spots size and location.

If type A abnormality on CC of OCTA was larger than CVH on ICGA as we found in previous study, OCTA-guided PDT may cover larger area than ICGA-guided PDT and may cause more severe in choriocapillaris ischemia. In this study, we report the choriocapillaris changes after half dose PDT. OCTA provide us a new weapon to observe choriocapillaris flow before and after PDT since its images were not affected by dye leakage and thus had the advantage to depict ischemia area which could be obscured in ICGA. Demircan reported choriocapillaris vessel density was significantly reduced within 1 week after PDT as compared with baseline [20]. Camilla reported a progressive significant reduction of the vascular component after PDT that last for 1 month [21], while Nassisi, Demirel both reported that choriocapillaris vessel density was higher than baseline at 1 month after PDT [22, 23]. Ho also report similar finding at 3-month follow-up after PDT [24]. Chen et al. reported that choriocapillary of CSC patients recovered to a relatively even appearance on OCTA at month 3 after PDT along with the absorption of SRF, but they did not do quantitative analysis [25]. In this study, choriocapillaris flow deficit was found in all study eyes which was consistent to many previous OCTA studies about CSC [26]. We also found the deficit area reduced at 1, 3, and 6 months after treatment with the trend of becoming more even on CC of OCTA in both groups. These findings on choriocapillaris changes following PDT provide us new evidence to support the mechanism of PDT which is remodeling choroidal choriocapillaris. However, we still need to pay attention on the fact that large amount of SRF may show artifact on CC and affect the interpretation of choriocapillaris deficit as we prescribed previously [9].

The limitations of this study were most related to the limitation of our SD-OCT equipment. First, the outer boundary of choroid could not be outlined reliably on our OCT and thus hindered our attempt to measure and compare the choroidal thickness between the two groups. Second, we only evaluated alteration on CC of OCTA because the Sattler and Haller layer did not show satisfactory imaging. Third, the FDD was calculated through the third-party software and cannot be compared directly to results from other publications using different measuring methods. Fourth, lack of follow-up within 1 week after PDT due difficulties during COVID-19 pandemic made it difficult to reveal CC changes shortly after PDT that can be transit. Fifth, we only include eyes with hyper-reflective areas on OCTA en face image at the CC level; the result of this study was not applicable to those CSC patients who do not show hyper-reflective areas on OCTA.

In summary, this 6-month randomized clinical trial proved the noninferiority of OCTA-guided PDT to ICGA-guide PDT for complete SRF resolution at 1 and 6 months after half dose PDT in the treatment of acute CSC. OCTA as a noninvasive imaging method may have the potential to replace ICGA as a tool for guidance and follow-up of PDT for acute CSC patients.
